# Preclinical evaluation of CXCR4 peptides for targeted radionuclide therapy in glioblastoma

**DOI:** 10.1186/s41181-024-00282-y

**Published:** 2024-07-15

**Authors:** Anthony Waked, Melissa Crabbé, Virginie Neirinckx, Sunay Rodriguez Pérez, Jasmien Wellens, Bernard Rogister, M. Abderrafi Benotmane, Koen Vermeulen

**Affiliations:** 1 Nuclear Medical Applications Institute, Belgian Nuclear Research Centre (SCK CEN), Mol, Belgium; 2 Laboratory of Nervous System Disorders and Therapy, GIGA Neurosciences, Université de Liège, Liège, Belgium; 3 Neurology Department, CHU de Liège, Liège, Belgium

## Abstract

**Background:**

Glioblastoma (GBM), is the most fatal form of brain cancer, with a high tendency for recurrence despite combined treatments including surgery, radiotherapy, and chemotherapy with temozolomide. The C-X-C chemokine receptor 4 (CXCR4) plays an important role in tumour radioresistance and recurrence, and is considered as an interesting GBM target. TRT holds untapped potential for GBM treatment, with CXCR4-TRT being a promising strategy for recurrent GBM treatment. Our study focuses on the preclinical assessment of different ^177^Lu-labelled CXCR4-targeting peptides, CTCE-9908, DV1-K-DV3, and POL3026 for GBM treatment and exploring some of the radiobiological mechanisms underlying these therapies.

**Results:**

All three DOTA-conjugated peptides could be radiolabelled with ^177^Lu with > 95% radiochemical yield. Binding studies show high specific binding of [^177^Lu]Lu-DOTA-POL3026 to U87-CXCR4 + cells, with 42% of the added activity binding to the membrane at 1 nM, and 6.5% internalised into the cells. In the presence of the heterologous CXCR4 blocking agent, AMD11070, membrane binding was reduced by 95%, a result confirmed by quantitative in vitro autoradiography of orthotopic GBM xenograft sections. An activity-dependent decrease in cell viability was observed for [^177^Lu]Lu-DOTA-DV1-K-DV3 and [^177^Lu]Lu-DOTA-POL3026, along with a slight increase in the induction of apoptotic markers. Additionally, the expression of γH2AX increased in a time-and activity-dependent manner. Ex vivo biodistribution studies with [^177^Lu]Lu-DOTA-POL3026 show uptake in the tumour reaching a SUV of 1.9 at 24 h post-injection, with higher uptake in the kidneys, lungs, spleen, and liver. Dosimetry estimations show an absorbed dose of 0.93 Gy/MBq in the tumour. A blocking study with AMD11070 showed a 38% reduction in tumour uptake, with no significant reduction observed in µSPECT imaging. Although no brain uptake was observed in the ex vivo biodistribution study, autoradiography on U87-CXCR4 + tumour inoculated mouse brain slices shows non-specific binding in the brain, next to high specific binding to the tumour.

**Conclusions:**

In conclusion, we compared different ^177^Lu-radiolabelled CXCR4-targeting peptides for their binding potential in GBM, and demonstrated their varied cytotoxic action against GBM cells in vitro, with POL3026 being the most promising, causing considerable DNA damage. Though the peptide’s systemic biodistribution remains to be improved, our data demonstrate the potential of [^177^Lu]Lu-DOTA-POL3026 for CXCR4-TRT in the context of GBM.

**Supplementary Information:**

The online version contains supplementary material available at 10.1186/s41181-024-00282-y.

## Introduction

Glioblastoma (GBM) is the most common primary brain tumour, with a median patient survival time ranging from 12 to 14 months (Witthayanuwat et al. 2018; Johnson and O’Neill 2012). Despite our increased understanding of the biology of this disease, only a few findings were translated into new clinical guidelines since the introduction of the Stupp’s protocol in 2005 (Kazda et al. 2018). This standard-of-care consists of maximal safe surgical resection, radiotherapy, and concomitant and adjuvant chemotherapy with temozolomide (TMZ). GBM recurrence rates are high, with limited treatment options after relapse, making clinical management of the recurrent disease challenging (Birzu et al. 2020). There is thus a need for new, effective therapies that can target GBM cells while sparing the surrounding brain tissue. A gamut of new therapies is being investigated at various levels of clinical development. Targeted radionuclide therapy (TRT), in particular, has generated a lot of interest within the oncology and nuclear medicine communities due to the recent clinical success of Luthathera^®^ ([^177^Lu]Lu-DOTATATE) for the treatment of gastroenteropancreatic neuroendocrine tumours (GEP-NETs) and Pluvicto^®^ ([^177^Lu]Lu-PSMA-617) for the treatment of prostate-specific membrane antigen (PSMA)-positive metastatic castration-resistant prostate cancer (mCRPC) (Hennrich and Eder 2022; Hennrich et al. 2019).

The success of targeted radionuclide therapies depends on a multitude of factors, the first of which is the identification of a clinical target with high expression in the patient tumour. C-X-C chemokine receptor type 4 (CXCR4) is a protein whose high expression in GBM tumours was found to be associated with shorter overall survival (OS) (Ma et al. 2017). Indeed, the protein plays an important role in GBM radioresistance through the facilitation of proneural-to-mesenchymal transition, which contribute to GBM cell invasion of the surrounding parenchyma and also through the role played by CXCR4 in the invasion of the subventricular zone by glioblastoma stem-like cells (GSCs), offering a ‘hideout’ from surgical intervention and conventional radiotherapy (Khan et al. 2023; Goffart et al. 2015, 2017; Lombard et al. 2020). CXCR4 is overexpressed in CD133 + GBM cells, which are often associated with GSCs, when compared with CD133- GBM cells. And GSCs are found to be enriched in recurring GBM tumours compared to the primary tumours of the same patients (Liu et al. 2006). The implication of CXCR4 in GBM therapy resistance and recurrence reinforces the potential benefit of targeting it to treat GBM recurrences.

The most clinically advanced CXCR4-targeting radiopharmaceuticals are the [^68^Ga]Ga-PentixaFor/[^177^Lu]Lu-PentixaTher theranostic analogues, initially developed for CXCR4-targeted imaging and treatment in hematological cancers, and now tested in several solid tumours (Vag et al. 2016). [^68^Ga]Ga-PentixaFor has also been used for CXCR4 imaging in GBM, highlighting its potential in the early detection of GBM recurrences and in assessing response to chemotherapy and radiotherapy (Waheed et al. 2024; Jacobs et al. 2022). However, to our knowledge, PentixaTher has not been used to treat GBM patients. There is a need to investigate and compare other CXCR4-targeting peptides to be used as potential carrier molecules for TRT in GBM.

**Fig. 1:**
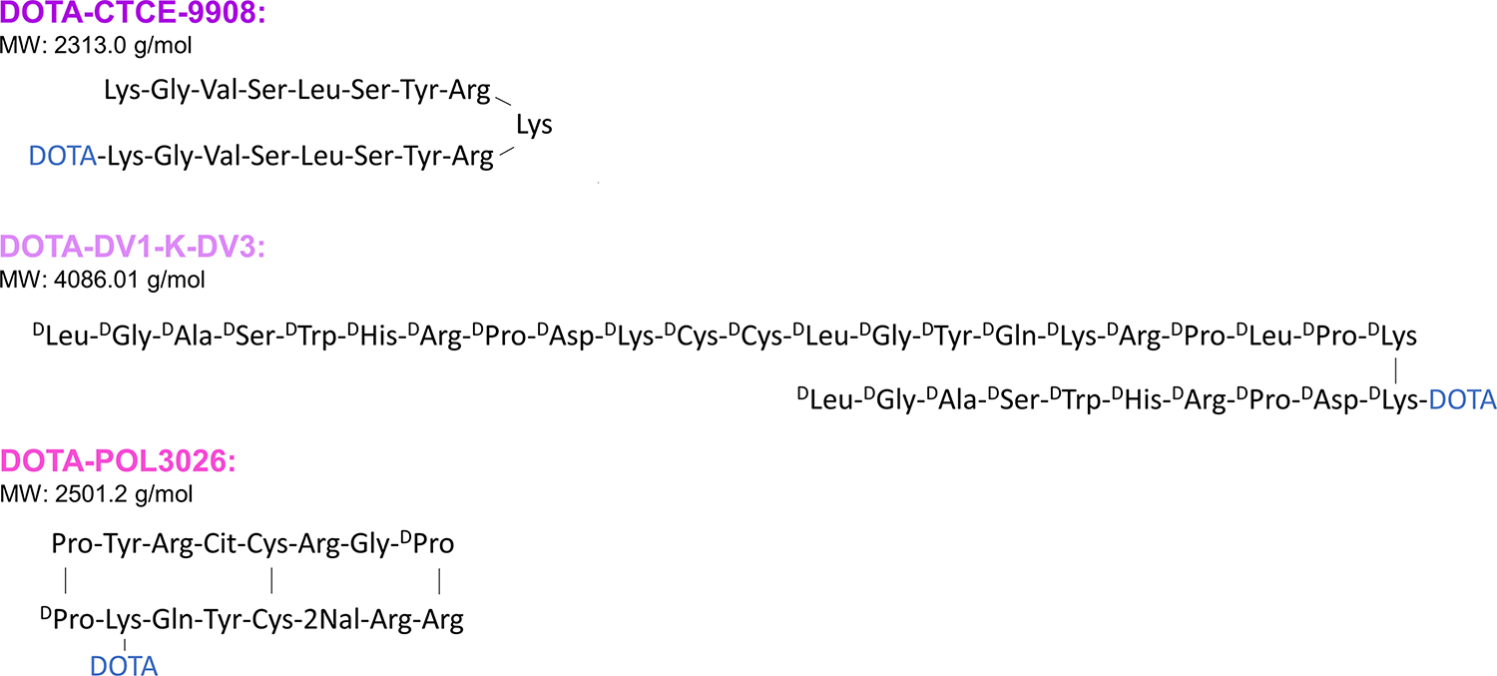
Structural amino acid sequence of the CXCR4-targeting peptides DOTA-CTCE-9908, DOTA-DV1-K-DV3, and DOTA-POL3026. D-amino acids are indicated with a superscript. MW = molecular weight, DOTA = 2,2′,2′′,2′′′-(1,4,7,10-Tetraazacyclododecane-1,4,7,10-tetrayl) tetraacetic acid

Three structurally-different peptides were used for this study: CTCE-9908, DV1-K-DV3, and POL3026 **(**Fig. 1**).** CTCE-9908 is a CXCR4 antagonist peptide made of a dimerised sequence of the disordered N-terminal region of CXCL12, the only known natural ligand of CXCR4 (Faber et al. 2007). To our knowledge, it has not been investigated as a theranostic agent before. The DVI-K-DV3 peptide was initially synthesized for its ability to inhibit HIV-1 infection through its strong antagonistic effect on CXCR4 (Xu et al. 2013). It has also been investigated for its potential in TRT by Luyten, et al. (Luyten et al. 2021). POL3026 is a cyclic peptide derived from polyphemusin-II analogues developed in 2006 and reported for its favorable pharmacokinetic properties and high CXCR4 selectivity (DeMarco et al. 2006). It has been further investigated by Lesniak, et al. as an ^111^In-based imaging agent in preclinical U87-CXCR4 + models (Lesniak et al. 2015a, b).

In this study, we aimed to preclinically evaluate three structurally-different ^177^Lu-radiolabelled CXCR4-targeting peptides for their potential as TRT in GBM. Specifically, we assessed their radiolabeling efficiency,  cell binding and internalisation, and effect on cell viability. Next, the most promising peptide was selected and further studied in vitro for its effects on DNA damage and cell death by apoptosis. Finally, in vitro autoradiography, ex vivo biodistribution, dosimetry estimations and µSPECT-CT imaging studies were performed to evaluate its uptake and pharmacokinetics.

## Materials and methods

### Peptides and chemicals

DOTA-CTCE9908, DOTA-DV1-K-DV3, and DOTA-POL3026 were obtained from the custom peptide synthesis service of CSBio (California, US), with quality control done using mass spectrometry and HPLC **(**Table 1**)**. They were dissolved in 0.15 M NaOAc, pH 4.7, aliquoted and stored at -20 °C. All compounds were used without further purification. AMD11070, a CXCR4 antagonist, was obtained from Adooq Biosciences (CN A11315, California, US). Other chemicals used are mentioned in their respective sections.

**Table 1:** Characterisation of the DOTA-conjugated peptides from CSBio

	DOTA-CTCE9908	DOTA-DV1-K-DV3	DOTA-POL3026
Product no.	CS9579	CS17790	CS17791
Lot no.	W152	W536	W490
Expected M.W. (g/mol)	2313.00	4086.01	2501.20
Found M.W. (g/mol)	2313.66	4088.00	2500.20
Purity (%)	98.07	95.08	96.14

### Cell culture

U87-MG cells overexpressing CXCR4 (U87-CXCR4+) (Sanchez Gil et al. 2022) were cultured at 37 °C and 5% CO_2_ in Dulbecco’s Modified Eagle Medium (DMEM) High Glucose, containing L-glutamine, and pyruvate (Gibco™, Fisher Scientific, Brussels, Belgium) and supplemented with 10% fetal bovine serum (FBS, Gibco™, Fisher Scientific, Brussels, Belgium). Blasticidin S (Sigma Aldrich^®^, Merck, Darmstadt, Germany) was also added to the complete media at a concentration of 0.5 mg/mL to maintain selective pressure for CXCR4 + cells. They were maintained up to a confluence of 80% before sub-culturing.

### Radiolabelling

All three CXCR4-targeting peptides were radiolabelled with ^177^Lu at a molar activity (A_m_) of 50 MBq/nmol. [^177^Lu]LuCl_3_ was added to the DOTA-conjugated peptides in 0.15 M NaOAc buffer, pH 4.7, with a total reaction volume of 0.25–0.35 mL, and reacted at 95 °C for 30 min with gentle shaking (Thermal Mixer, Thermo Fischer Scientific, Massachusetts, US). The radiolabeling yield was evaluated by spotting 2 µL of the radiolabelled solution on a glass microfiber chromatography paper strip impregnated with silica gel (iTLC-SG, Agilent Technologies, Diegem, Belgium) that was eluted with a 0.5 M citrate solution (pH 5.5). After elution, the iTLC papers were cut into two segments and the activity of the bottom and top parts of the paper was counted for 2 min each using an automated gamma counter (2480 Wizard^2^, Perkin Elmer, Zaventem, Belgium). Colloid formation was assessed in a similar manner by performing a mock radiolabeling, applying similar conditions but omitting the peptides.

### Radiochemical stability

The stability of the radiolabelled peptides was evaluated in different physiologically relevant media. Around 10 MBq of the radiolabelled peptides (50 µL) was added to 150 µL of PBS, FBS or DMEM media, and incubated at 37 °C for 7 days. A 2 µL sample was spotted on the iTLC system described above to identify the intact fraction.

### Cell binding and internalisation assays

In the saturation binding assays, U87-CXCR4 + cells were seeded in 24-well plates and allowed to adhere. The night before the experiment, complete media was replaced with media containing 2% FBS. To determine the total binding, cells were treated with a mixture of each radiolabelled peptide (at final concentrations ranging from 0.1 to 1000 nM, corresponding to activity concentrations ranging from 0.005 to 50 MBq/mL) with 0.2% dimethyl sulfoxide (DMSO, vehicle). Non-specific binding (NSB) was verified by treating cells with a mixture of each radiolabelled peptide with 100 µM of AMD11070, a potent CXCR4 antagonist.

After 4 h of incubation at 37 °C and 5% CO_2_, the unbound fraction was collected in gamma counter tubes, and the cells were washed twice with PBS. The wash fractions were added to the same gamma counter tubes. Next, cells were lysed by incubation with 1 M NaOH for 30 min at room temperature (RT). After collecting the lysed cell fractions, two washes with 1 M NaOH were performed, and added to the same fractions, corresponding to the total bound fractions.

For the internalisation assay, 1 and 100 nM of the radiolabelled peptides were used. The membrane-bound fraction was separated from the internalised fraction by a 10 min incubation at RT with a stripping buffer of 50 mM glycine and 100 mM NaCl, pH 2.8. This was followed by lysis with 1 M NaOH as described above.

The activities of all collected fractions were measured in an automated gamma counter. Activity measurements were converted to % added activity (%AA) and normalised for cell counts. A standard curve, obtained by measuring the treatment solutions, was used to convert the activity concentration to moles.

### MTS cell viability assay

Cell viability assays were performed using increasing activity concentrations of the radiolabelled peptides (1 to 20 MBq/mL), incubated with U87-CXC4 + cells for four hours at 37 °C, 5% CO2. After the incubation period, cells were washed twice with PBS and replenished with complete media. After five and seven days of treatment, the cell media was removed and MTS (3-(4,5-dimethylthiazol-2-yl)-5-(3-carboxymethoxyphenyl)-2-(4-sulfophenyl)-2 H-tetrazolium) reagent added, prepared according to manufacturer’s instructions (CellTiter 96^®^ AQueous One Solution Cell Proliferation Assay, Promega, Wisconsin, United States). To determine the effect of the radiation originating from unbound radiolabelled peptides, cells were treated with equivalent activity concentrations of [^177^Lu]Lu-DTPA. Triton X-100 (0.1%) was used as a positive control. Vehicle includes the NaOAc radiolabeling buffer at the concentration used for the 20 MBq/mL peptide solutions. The cells were incubated for 0.5–1 h with the MTS reagent at 37 °C, 5% CO_2_. Absorbance was measured at 490 nm using a BioTek Synergy H1 plate reader (Agilent Technologies, Mechelen, Belgium). Cell viability was normalised to the vehicle and calculated as percentage of cell viability. Results show the means of 3–4 independent experiments run in triplicates.

### Apoptosis live cell imaging

Apoptosis was measured using the IncuCyte^®^ Zoom system (Essen Bioscience, Hertfordshire, UK).

Briefly, cells were seeded in 96-well plates, and left to attach overnight at 37 °C, 5% CO_2_. Next, cells were treated with different activity concentrations of [^177^Lu]Lu-DOTA-POL3026 or [^177^Lu]Lu-DTPA, ranging from 1 to 20 MBq/mL. After four hours of incubation at 37 °C and 5% CO_2_, cells were washed twice with PBS. The Sartorius™ IncuCyte^®^ dyes Caspase 3/7 green reagent and Annexin V red reagent were added at 1/2000 and 1/200 dilutions, respectively. Images were analysed using the IncuCyte^®^ Zoom software. The average values of Green Calibrated Unit GCU x µm² (for Caspase 3/7) and Red Calibrated Units RCU x µm² (for Annexin V) signal were normalised to cell confluence percentages, five and seven days after treatment. Vehicle includes the NaOAc radiolabeling buffer at the concentration used for the 20 MBq/mL peptide solutions. Results show the means of two independent experiments, run in triplicates.

### DNA damage immunofluorescence

DNA damage in U87-CXCR4 + cells was detected by immunofluorescent staining of the DNA damage and repair proteins, phospho-H2A histone family member X (yH2Ax) and p53-binding protein 1 (53BP1). Briefly, cells were seeded in 96-well plates and left overnight to adhere at 37 °C, 5% CO_2_. Next, cells were treated with different activity concentrations of [^177^Lu]Lu-DOTA-POL3026 or [^177^Lu]Lu-DTPA, ranging from 1 to 20 MBq/mL. After four hours of incubation, cells were washed twice with PBS, replenished with fresh media, and left at 37 °C, 5% CO_2_ until the fixation time point. For fixation, cells were washed with PBS, and fixed by incubating them with 4% paraformaldehyde (PFA) for 15 min at RT. This was done 0, 24 and 120 h after treatment. Cells were washed twice with PBS and stored in PBS at 4 °C until the residual radioactivity in the plate decayed. The vehicle includes the NaOAc radiolabeling buffer at the concentration used for the 20 MBq/mL peptide solutions.

After decay storage, yH2Ax/53BP1 double staining was performed using the following protocol. First, cells were washed once with PBS-glycine and then permeabilised by washing them for 10 min with PBS-Triton X-100. Blocking was performed using 5% bovine serum albumin (BSA) for 1 h at RT. Cells were then incubated for 1 h at 37 °C with mouse monoclonal anti-yH2Ax antibody at 1:500 dilution (05-636, Merck Millipore, Brussels, Belgium) and rabbit polyclonal anti-53BP1 antibody at 1:5000 dilution (NB100-304). Cells were incubated for 1 h at 37 °C with Alexa fluor 568 goat anti-mouse and Alexa fluor 488 goat anti-rabbit antibodies diluted at 1:500 and 1:1000, respectively, in 5% BSA. Finally, cells were mounted using Ibidi mounting medium with DAPI (50,011, Ibidi, Gräfelfing, Germany). Each step was preceded by three washes with PBS-glycine.

Cells were imaged at 40x magnification using a BioTek Cytation 5 (Agilent Technologies, Mechelen, Belgium), where 4 fields were imaged per well, ensuring that at least a hundred cells were counted per condition. Analysis was performed by the Cytation 5 software and the object count (number of DAPI stained nuclei), Green Fluorescent Protein (GFP) spot count (corresponding to the average number of yH2Ax foci per nucleus) and Red Fluorescent Protein (RFP) spot count (corresponding to the average number of 53BP1 foci per nucleus) were measured and normalised to the spot counts of the vehicle condition. Results show the means of two independent experiments, run in at least triplicates.

### Generation of xenograft mice models

All animal experiments were performed in compliance with the Ethical Committee, Animal Studies of Medanex Clinic (EC MxCl 2023 − 218), the Belgian laboratory animal legislation and the European Communities Council Directive of 22 September 2010 (2010/63/EU). Six-week old female BALB/cAnNRj-Foxn1 nu/nu mice were ordered from Janvier (Bio Services, Uden, The Netherlands) and housed in ventilated cages under standard laboratory conditions (12 h light/dark cycle) at the animal facility of SCK CEN. All animals had access to food and water *ad libitum.*

For the generation of the subcutaneous GBM xenograft model, BALB/c nude mice (between 8 and 12 weeks old) were anesthetised with 2.5% isoflurane in O_2_ at a flow rate of 0.2 L/min and subcutaneously injected on the right shoulder with 3 × 10^6^ U87-CXCR4 + cells resuspended in a 1:1 mixture of PBS and Cultrex BME (R&D Systems, Bio-Techne, Minnesota, US). Tumours were left to grow for ten days before the start of the experiment. Tumour volumes were calculated as length × width × height. Three days before the experiment, the mean tumour volumes of all mice were 255 ± 119 mm^3^.

The protocol for the development of orthotopic U87-CXCR4 + xenografts in the mouse brain, from which sections were used for our in vitro autoradiography study, has been previously reported (Sanchez Gil et al. 2022).

### In vitro autoradiography

Mice were sacrificed by an overdose of pentobarbital (200 µL of 60 mg/mL), followed by perfusion with saline via the left ventricle. Organs of interest (including tumour and spleen) were quickly excised, rinsed with PBS, and rapidly frozen in 2-methylbutane (-40 °C). Slices (14 μm) were acquired by cryotome sectioning (Cryostar™ NX50, Thermo Fisher Scientific, Brussels, Belgium), fixed on Superfrost™ Plus Adhesion Microscope Slides (Epredia, Breda, Netherlands) and stored at -20 °C until further use. A blocking study was conducted on U87-CXCR4 + tumour sections (either subcutaneously grown or inoculated in brain) and on mouse spleens as positive control. Additionally, a saturation binding study was performed to determine the affinity (K_D_) and maximal number of receptors (B_max_) on U87-CXCR4 + tumour slices. The buffers used in both experiments are listed in Table 2.

Frozen slides were first air-dried and then submerged in buffer A for 30 min at RT, to remove endogenous ligands. Next, slides were dried at low heat with a heat gun. After complete dryness, slides were incubated for 60 min at RT with 500 µl of:

- Blocking study: 2.5 or 250 nM [^177^Lu]Lu-DOTA-POL3026 at 50 MBq/nmol in the presence of vehicle or 100 µM AMD11070, dissolved in buffer B, to determine binding specificity.
- Saturation binding study: a concentration range (0.1–2000 nM) of [^177^Lu]Lu-DOTA-POL3026 at 50 MBq/nmol with vehicle or 100 µM AMD11070 dissolved in buffer B, to respectively determine total and NSB.

After incubation, the solutions were removed, and slides were washed three times in buffer C for 10 min on ice. This was followed with a 5 min wash in milliQ H_2_O on ice to remove buffer salts. Slides were dried again at low heat with a heat gun. Slides were exposed to a phosphor storage screen (HR imaging plate, Elysia S.A. Liège, Belgium) for 2–60 min (depending on activity level) after which they were read with a CR-35 Bio high sensitivity image plate scanner (Elysia S.A., Liège, Belgium). Autoradiograms were analysed using the Aida image analyser software (v.5.1, Elysia S.A., Liège, Belgium). The percentage block was calculated as (1 - (average intensity/mm^2^ in the presence of 100 µM AMD11070)) / (average intensity/mm^2^ control conditions) x 100% on 3 tissue sections, depicted as mean ± SD. For the saturation binding experiment, the read-out data, intensity, was converted to pmol via a standard curve obtained from measuring a strip spotted with 2 µL of the different incubation solutions. The K_D_ (nM) and B_max_ (fmol/mm^2^) values were calculated using a non-linear regression model in GraphPad Prism 10.

**Table 2:** Buffers used during each step of the in vitro autoradiography protocol

Buffer	Step	Buffer constitution
A	Pre-incubation	HEPES 50 mM, pH 7.4, MgCl_2_ 5 mM, EDTA 1 mM, NaCl 100 mM + 1% BSA
B	Incubation	HEPES 50 mM, pH 7.4, MgCl_2_ 5 mM, EDTA 1 mM, NaCl 100 mM + 1% BSA + 40 µg/mL bacitracin
C	Washing	HEPES 50 mM, pH 7.4, MgCl_2_ 5 mM, EDTA 1 mM, NaCl 100 mM + 0.25% BSA

### Ex vivo biodistribution

The pharmacokinetic profile of [^177^Lu]Lu-POL3026 was evaluated in female BALB/c nude mice with subcutaneous xenografts of U87-CXCR4 + tumours.

All animals were anesthetised with 2.5% isoflurane in O_2_ at a flow rate of 1 L/min and intravenously (IV) injected with 1.5 MBq of [^177^Lu]Lu-POL3026 via a tail vein (radiolabelled with a A_m_ of 53.1 ± 0.55 MBq/nmol). The mice were sacrificed by an overdose of pentobarbital (200 µL of 60 mg/mL) at 1-, 4-, 24- and 168 h post-injection (p.i.) (*n* = 3–4 per time point), followed by cardiac puncture. Organs of interest were collected in tarred tubes and weighed. The activity in the different organs was measured in the automated gamma counter described above. For the calculation of the total activity in the blood, bone, and muscle, the masses were assumed to be 7, 12 and 40% of the total body mass, respectively. Data is shown as standardised uptake value (SUV), calculated using the following equation:

SUV = [Activity in organ (cpm) / Organ weight (g)] / [(Injected activity (cpm) / Total body weight (g)].

A separate study was performed to evaluate the specificity of the uptake of [^177^Lu]Lu-POL3026 in U87-CXCR4 + subcutaneous tumours and the rest of the organs. This was done by creating two groups (*n* = 4 / group), one group pre-injected intraperitoneally (IP) with 5 mg/kg of AMD11070, 30 min before the IV injection of [^177^Lu]Lu-POL3026, while the other group was pre-injected with the DMSO vehicle. Mice from both groups were sacrificed 4 h post-injection and organs were collected as previously described.

### Dosimetry estimation

Dosimetry calculations were performed for the organs that showed higher uptake (i.e. kidneys, liver and spleen) and the tumour using the Medical Internal Radiation Dose (MIRD) formalism (Bolch et al. 2009). The counts per minute (cpm) data from the ex vivo biodistribution study were converted to activity units using the gamma counter response curve (cpm/MBq vs. A(MBq) for Lu-177. The response curve was derived from measurements over time of samples with a known activity concentration traceable to a secondary standard ionisation chamber (Fidelis, Southern-Scientific, Henfield, UK).

To obtain the time activity curve (TAC), the fraction of injected activity per gram of dissected tissue (FIA/g) was plotted as a function of time. A linear increase of the FIA/g from zero at t = 0 h until t = 1 h (first measured time point) was assumed. The activity values were decay corrected to the time of sacrifice of each mouse. The TACs were then fitted to a bi-exponential function with R^2^ values of 0.93, 0.98, 0.99 and 0.99 for spleen, tumor, kidney and liver respectively. The area under the curve (AUC) was then obtained by integrating the TACs from zero to infinity. Then, the time-integrated activity coefficient (ã) was obtained by multiplying the mean tissue mass (i.e., m = 0.16 g for tumour, m = 0.30 g for kidneys, m = 1.16 g for liver and m = 0.07 g for the spleen) by the respective AUC.

Following the MIRD equation, the mean absorbed dose for the target tissue was obtained by multiplying ã in the source tissue by the absorbed dose (in the target tissue) per unit cumulated activity in the source tissue (i.e., S value). The S-values were calculated using OLINDA/EXM software (Hermes Medical Solutions) (Stabin and Siegel 2018). The sphere model was used to calculate the S value for the tumour with a mass of 0.16 g. For the kidney, liver and spleen, the 25 g mouse model was used, and the organs masses were adjusted to the respective mean mass of the dissected organs. ^177^Lu dose coefficients for the self-dose for tumor, kidney, liver and spleen were 0.41, 0.076, 0.02 and 0.32 mGy/MBq∙s respectively.

### µSPECT-CT imaging

µSPECT-CT imaging was performed at 4 and 24 h post-injection using a dedicated small-animal µSPECT-CT imaging system (U-SPECT^6^-CT, MILabs, Houten, The Netherlands). Tumour-bearing mice (mean tumour volume: 260 ± 35 mm^3^) were pre-treated IP with either AMD11070 (5 mg/kg; *n* = 2) or DMSO vehicle (*n* = 2) 30 min pre injection of [^177^Lu]Lu-POL3026. µSPECT images were acquired in list mode with the GP-M collimator for approximately 80 min following injection of 27.50 ± 0.46 MBq of [^177^Lu]Lu-POL3026 (*n* = 4, radiolabelled with a A_M_ of 54.0 MBq/nmol). Mice were maintained under anaesthesia (2.5% isoflurane, 1 L/min O_2_ as the carrier gas) during image collection.

Reconstruction of the µSPECT images was performed using the similarity-regulated ordered subsets expectation maximization (SROSEM) algorithm, with four iterations, 128 subsets and a voxel size of 0.4 mm. Two photopeak windows of 206 keV (± 18%) and 115 keV (± 18%) were utilised for image reconstruction. Scatter correction was applied using the triple energy window method. µSPECT images were registered to the CT images and corrected for photon attenuation based on the CT data. The calibration factor for conversion to MBq/cc was determined through imaging of an Eppendorf tube filled with ~ 0.5 mL of 4.5 MBq [^177^Lu]LuCl_3_ (activity measurement was performed in a calibrated activity calibrator). The displayed images were converted to SUV based on the formula described above where the activity measured from an image acquired at time t, decay corrected to t = 0 (injection time) and expressed as volume concentration (e.g. MBq/cc) and injected activity is the activity at t = 0 in MBq. Fiji and MIPAV software were used for image analysis.

### Statistical analysis

Statistical analyses and data plotting were done using GraphPad Prism 10. Data from saturation binding experiments on cells and autoradiography tumour slices were plotted using non-linear regression to determine K_D_ values. Statistical significance was evaluated with one-way ANOVA followed by post-hoc comparisons with the untreated cells using Dunnett’s test. For data that are non-normally distributed, Kruskal-Wallis test was used to determine statistical significance. The number of independent experiments performed is indicated under each figure (N = X). Each condition within an experiment has been replicated at least three times (*n* = 3+).

## Results

### Radiochemical stability of the radiolabelled peptides

**Fig. 2:**
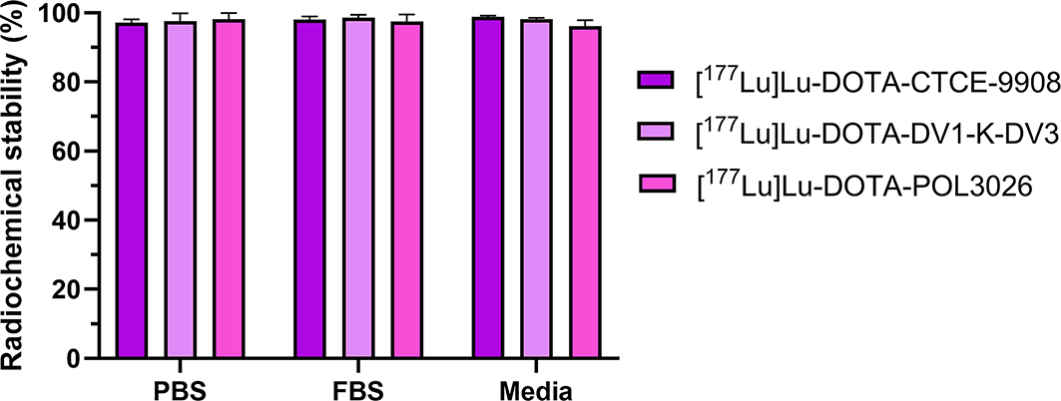
Stability of [^177^Lu]Lu-DOTA-CTCE9908, [^177^Lu]Lu-DOTA-DV1-K-DV3, and [^177^Lu]Lu-DOTA-POL3026 7 days after incubation at 37 °C in PBS, FBS, and DMEM media. Data express mean ± SD (*N* = 3)

The radiolabelling of all three DOTA-conjugated peptides consistently resulted in high radiochemical yields (> 95%) at a A_m_ of 52.95 ± 1.59 MBq/nmol. Colloid formation amounted to 2.6%.

To evaluate the radiochemical stability of the radiolabelled peptides in different solutions, we incubated them at 37 °C in PBS, FBS, and DMEM media and observed no considerable dissociation of ^177^Lu from the DOTA-peptide complexes up to seven days post-radiolabelling (> 95%), as determined by iTLC **(**Fig. 2**).**

### Binding characteristics of the radiolabelled peptides

**Fig. 3:**
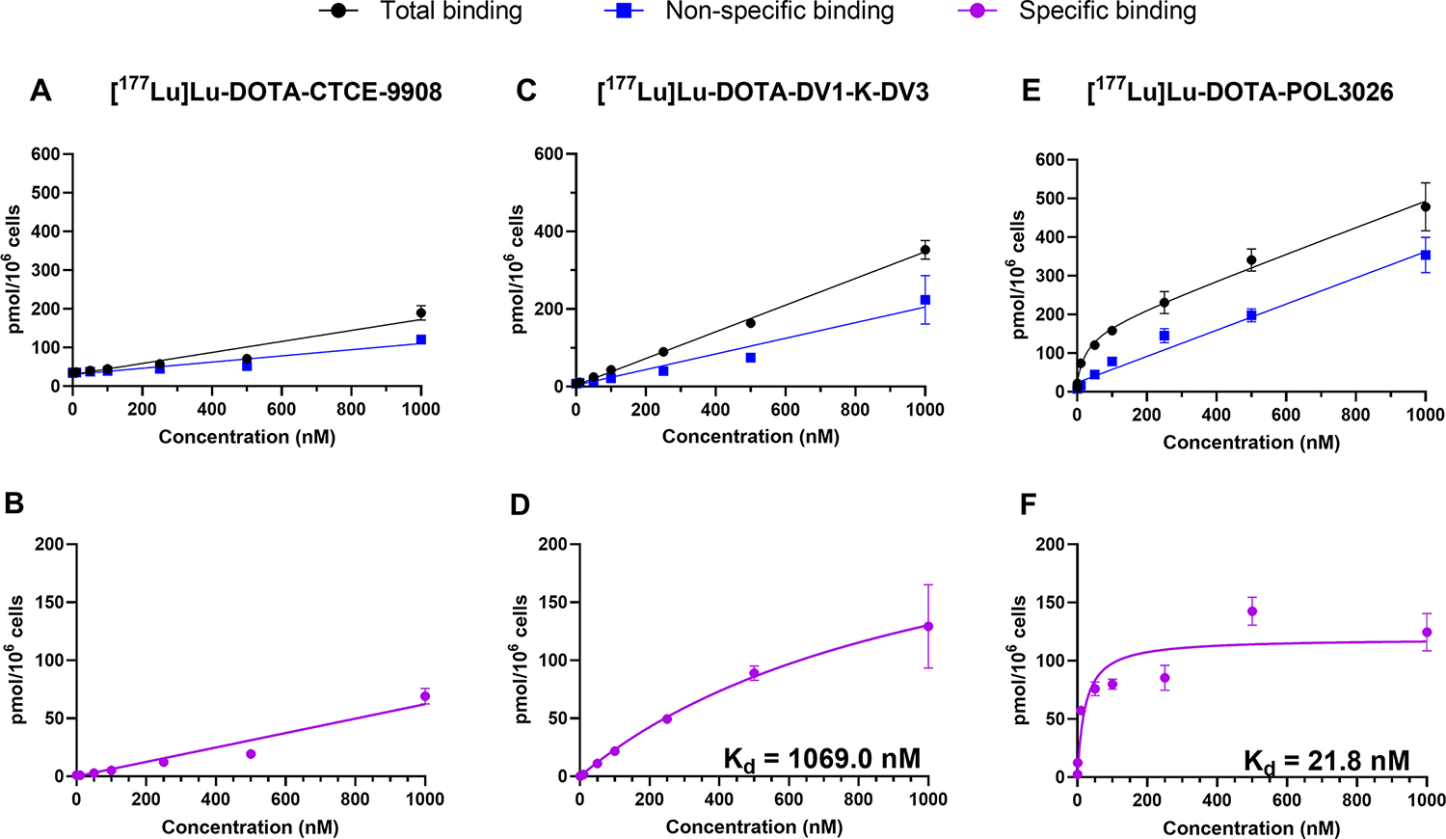
Total binding and non-specific binding curves of [^177^Lu]Lu-DOTA-CTCE9908 (**A**), [^177^Lu]Lu-DOTA-DV1-K-DV3 (**B**), and [^177^Lu]Lu-DOTA-POL3026 (**C**) as determined from a saturation binding assay in U87-CXCR4 + cells. Results are expressed as pmol/10^6^ cells, and expressed as mean + SD (*N* = 2)

In order to determine the affinity of the peptides to CXCR4, saturation binding assays were performed on U87-CXCR4 + cells, with peptide concentrations ranging from 0.1 nM to 1000 nM of the radiolabelled peptides **(**Fig. 3**).** We could not calculate the affinity values (K_D_) for [^177^Lu]Lu-DOTA-CTCE9908 due to its uptake not reaching saturation within the concentration ranges tested, reflecting a low specific binding. [^177^Lu]Lu-DOTA-DV1-K-DV3 exhibited a K_D_ of 1069.0 nM, and [^177^Lu]Lu-DOTA-POL3026 exhibited a K_D_ of 21.8 nM.

**Fig. 4:**
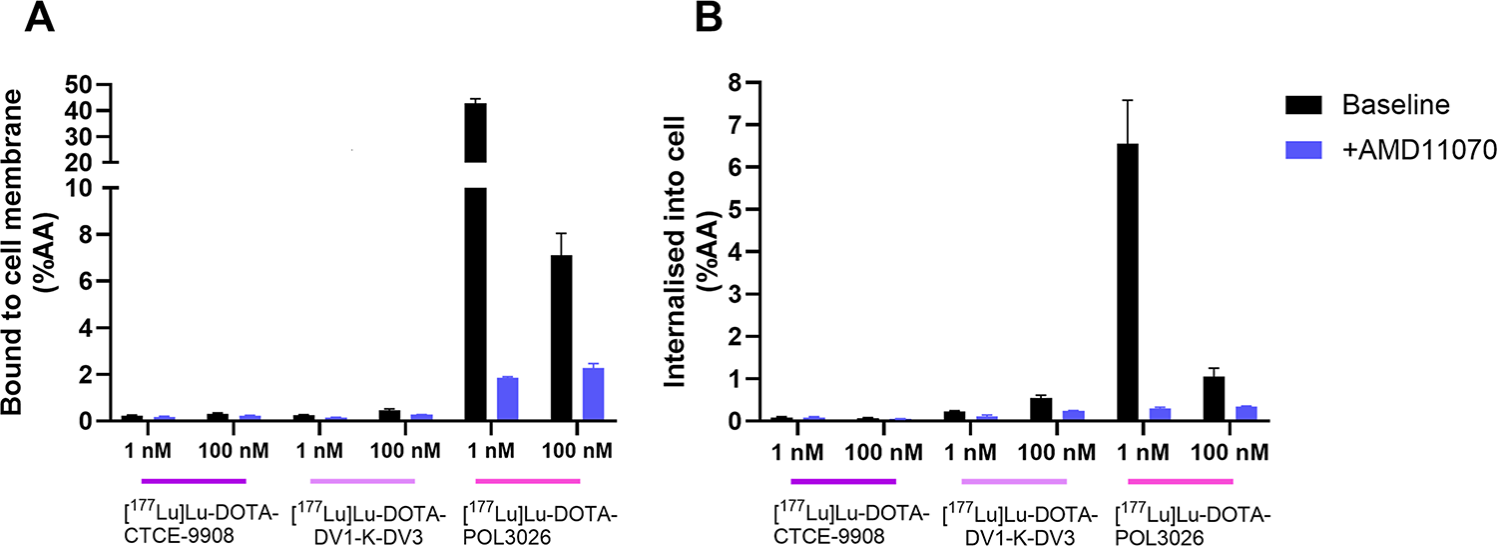
Percentage of added activity bound to the U87-CXCR4 + cell membrane (**A**) or internalised (**B**) at 1 nM and 100 nM after incubation with *[*^*177*^*Lu]Lu-DOTA-CTCE9908*,* [*^*177*^*Lu]Lu-DOTA-DV1-K-DV3* (**B**), * and [*^*177*^*Lu]Lu-DOTA-POL3026. Data are expressed as mean ± SD* (*N* = 2)

In an uptake experiment (Fig. 4), U87-CXCR4 + cells were treated with 1 or 100 nM of one of the three radiolabelled peptides. Both [^177^Lu]Lu-DOTA-CTCE9908 and [^177^Lu]Lu-DOTA-DV1-K-DV3 showed less than 1 %AA associated with the cell membrane at 1 nM, while 42.7 ± 1.8% of [^177^Lu]Lu-DOTA-POL3026 was bound. More so, [^177^Lu]Lu-DOTA-POL3026 binding was highly specific, indicated by a decrease to 1.9 ± 0.1% in the presence of 100 µM AMD11070. At 1 nM, 0.08 ± 0.02, 0.2 ± 0.02, and 6.6 ± 1.0 %AA was internalised for [^177^Lu]Lu-DOTA-CTCE9908, [^177^Lu]Lu-DOTA-DV1-K-DV3, and [^177^Lu]Lu-DOTA-POL3026, respectively. Again, high specific binding was observed for [^177^Lu]Lu-DOTA-POL3026 (> 95% reduction of binding in presence of 100 µM AMD11070) while this was negligible for [^177^Lu]Lu-DOTA-CTCE9908 and [^177^Lu]Lu-DOTA-DV1-K-DV3. Increasing the incubation concentration to 100 nM did not significantly increase the uptake of [^177^Lu]Lu-DOTA-CTCE9908 or [^177^Lu]Lu-DOTA-DV1-K-DV3. The uptake of [^177^Lu]Lu-DOTA-POL3026, presented as %AA, was decreased at this higher concentration.

### Effect of the radiolabelled peptides on cell viability

**Fig. 5:**
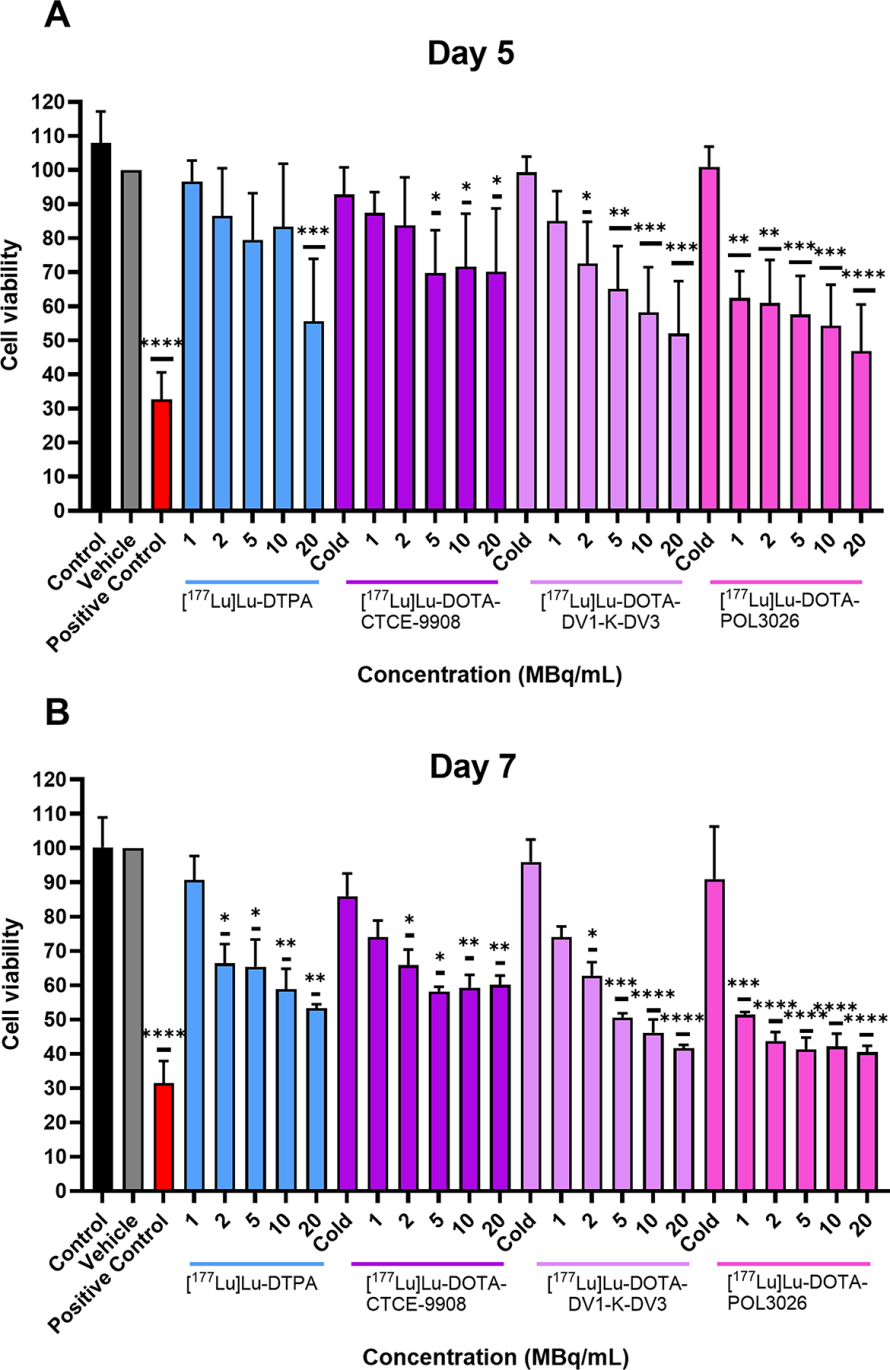
U87-CXCR4 + cell viability, measured with an MTS assay, at day 5 (**A**) and day 7 (**B**) after the incubation of the radiolabelled peptides and [^177^Lu]Lu-DTPA for 4 h. Data are expressed as a mean percentage of the vehicle-treated cells ± SD, with asterisks indicating statistically significant difference compared to the vehicle condition (*p < 0.05,**p ≤ 0.01,***p ≤ 0.001,****P ≤ 0.0001, *N* = 3–4)

In order to measure any therapeutic effect of the radiolabelled peptides in vitro, we conducted cell viability experiments with MTS, measuring viability at 5 and 7 days post-treatment, as shown in Fig. 5.

At day 5 post-treatment, [^177^Lu]Lu-DOTA-POL3026 exhibits a statistically significant decrease in cell viability (compared to control) at all activity concentrations of 1, 2, 5, 10, and 20 MBq/mL, resulting in cell viability ranging from 57.5 ± 4.3% to 42.5 ± 7.3% while treatment with [^177^Lu]Lu-DOTA-DV1-K-DV3 results in a statistically-significant, and activity-dependent decrease in cell viability starting from 2 MBq/mL at 64.4 ± 4.3% reaching a viability of 47.3 ± 8.2% at 20 MBq/mL. [^177^Lu]Lu-DOTA-CTCE9908 only causes a statistically significant reduction of cell viability at 5–20 MBq/mL, where it ranges from 65.2 ± 5.6% – 62.7 ± 13.3%. [^177^Lu]Lu-DTPA, on the other hand only causes a reduction in cell viability at 20 MBq/mL (50.2 ± 10.4%). It is interesting to note that at day 7, [^177^Lu]Lu-DTPA causes a statistically-significant reduction in cell viability starting from 2 to 20 MBq/mL, showing the significant contribution of the free activity in the medium at later timepoints. Viability at 1 MBq/mL was 90.9 ± 7.8%, 75.2 ± 15.1%, 75.2 ± 14.3%, and 52.3 ± 9.9% for [^177^Lu]Lu-DTPA, [^177^Lu]Lu-DOTA-CTCE9908, [^177^Lu]Lu-DOTA-DV1-K-DV3, and [^177^Lu]Lu-DOTA-POL3026, respectively.

In light of the preliminary in vitro data obtained, it was decided to continue with [^177^Lu]Lu-DOTA-POL-3026 for further evaluations.

### Effect of [^177^Lu]Lu-DOTA-POL3026 treatment on apoptosis

**Fig. 6:**
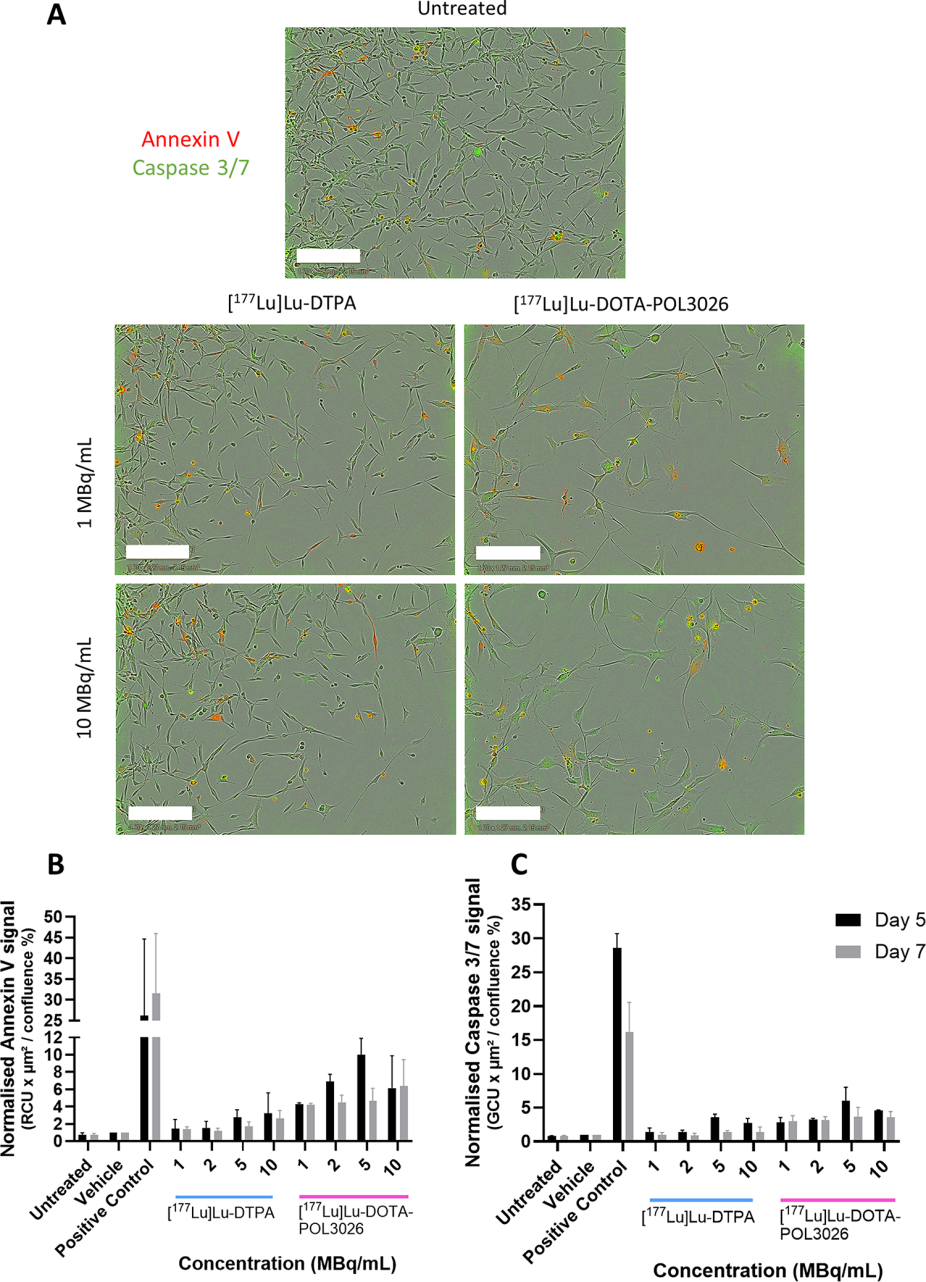
Live cell images of U87-CXCR4 + cells incubated with Annexin V (red) and Caspase3/7 (green) dyes 5 days post-treatment with 1 or 10 MBq/mL of [^177^Lu]Lu-DTPA and [^177^Lu]Lu-DOTA-POL3026. Untreated cells are also shown. Scale bar = 300 μm (**A**). Quantification of apoptosis markers Annexin V (**B**) and Caspase 3/7 (**C**) in cells at day 5 and day 7 post-treatment for 4 h (1–20 MBq/mL). Results are reported as a combined measure of fluorescence intensity and area, divided by cell confluence, and normalised to the vehicle condition. No statistically significant difference with the vehicle (*N* = 2) was observed.

A live cell imaging study was conducted comparing [^177^Lu]Lu-DOTA-POL-3026 and [^177^Lu]Lu-DTPA for their potential to induce apoptosis. Cells were exposed to radioactivity for 4 h, washed and incubated with annexin V and caspase 3/7 dyes (Fig. 6). Annexin V and caspase 3/7 signals for each condition were normalised to cell confluence and to the vehicle condition. At day 5, the annexin V signal for [^177^Lu]Lu-DTPA ranged from 1.4 ± 0.8 times the vehicle at 1 MBq/mL up to 3.3 ± 3.3 at 10 MBq/mL, whereas for [^177^Lu]Lu-DOTA-POL3026, the signal ranged from 4.3 ± 0.2 at 1 MBq/mL to 9.9 ± 2.7 at 5 MBq/mL. On day 7, Annexin V signals were reduced for most conditions tested.

Caspase 3/7 signals at day 5 ranged from 1.4 ± 0.8 at 1 MBq/mL to 2.8 ± 0.9 at 10 MBq/mL of [^177^Lu]Lu-DTPA, compared to 2.8 ± 0.9 at 1 MBq/mL to 6.0 ± 2.9 at 5 MBq/mL for [^177^Lu]Lu-DOTA-POL3026.

It should be noted, however, that none of the tested conditions amounted to a statistically significant increase compared to the vehicle condition.

### Effect of [^177^Lu]Lu-DOTA-POL3026 treatment on DNA damage

**Fig. 7:**
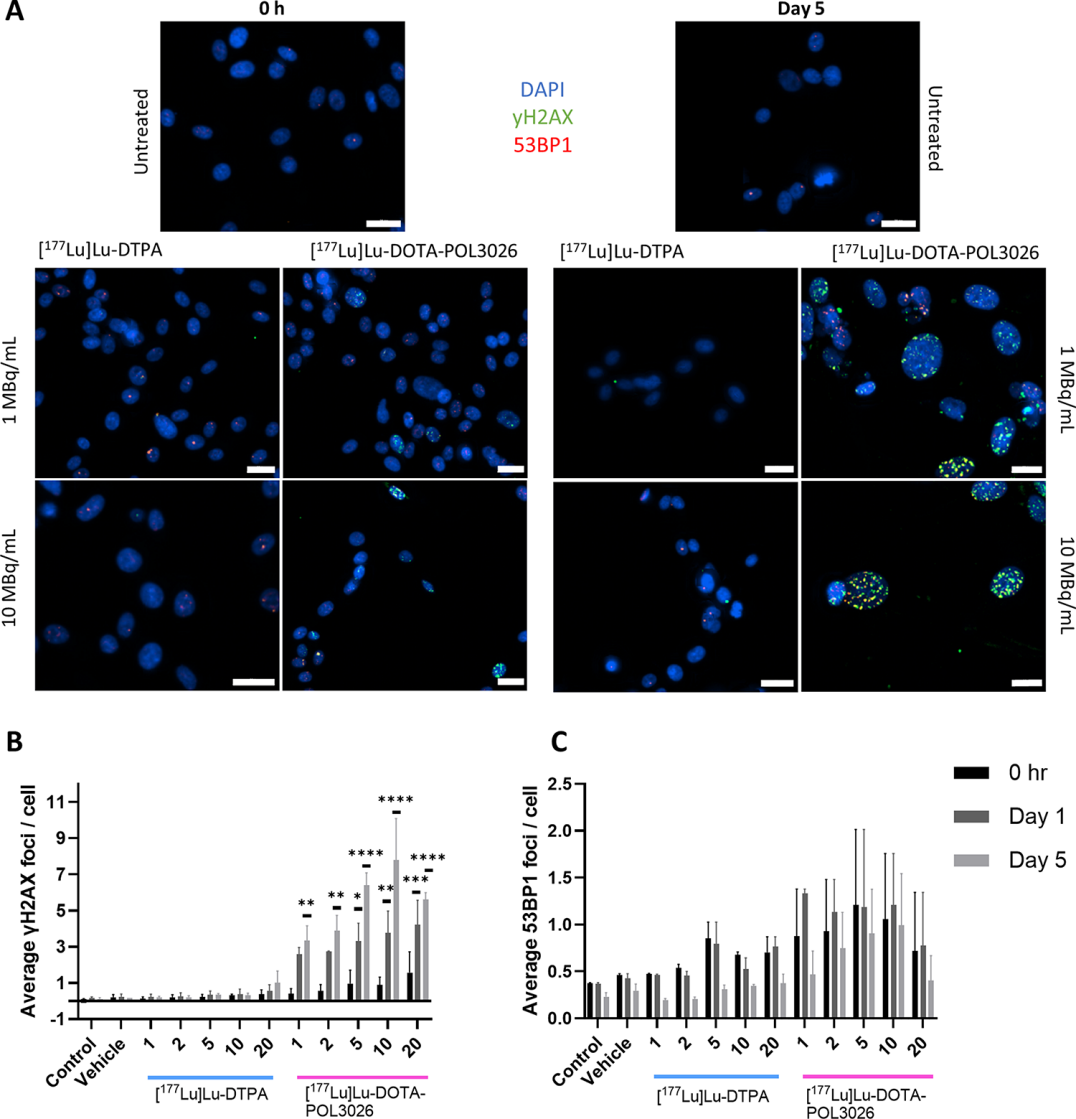
Microscopic images of immunostainings of yH2AX and 53BP1 in U87-CXCR4 + cell nuclei treated with 1 or 10 MBq/mL of [^177^Lu]Lu-DTPA and [^177^Lu]Lu-DOTA-POL3026 immediately after treatment and at day 5. (**A**). Quantification of yH2AX (**B**) and 53BP1 (**C**) expressed as average number of foci/cell in cells at 0 h, 1 day, and 5 days post-treatment. Scale bar = 20 μm. Asterisks indicate statistically significant difference with the vehicle condition (*p < 0.05,**p ≤ 0.01,***p ≤ 0.001,****P ≤ 0.0001). Data depicted as mean ± SD (*N* = 2)

To test the effects of [^177^Lu]Lu-DOTA-POL-3026 and [^177^Lu]Lu-DTPA on DNA damage, immunofluorescent imaging of DNA damage and repair proteins yH2AX and 53BP1 was performed (Fig. 7). Data was acquired 0 h, 1 day, and 5 days post-treatment. The results indicate a statistically-significant increase in yH2AX proteins at day 5 for all activity concentrations of [^177^Lu]Lu-DOTA-POL3026 (1–20 MBq/mL) in an activity-dependent manner. At 1 MBq/mL, an average of 3.3 ± 1.2 foci/cell were counted, increasing to 7.8 ± 3.2 foci/cell at 10 MBq/mL. On day 1 post-treatment, yH2Ax proteins only significantly increased at [^177^Lu]Lu-DOTA-POL3026 concentrations of 5 MBq/mL and higher. Treatment with [^177^Lu]Lu-DTPA did not result in an increase of yH2AX at any of the activity concentrations. No statistically-significant increase in 53BP1 was observed at any timepoint after treatment with [^177^Lu]Lu-DOTA-POL3026 or [^177^Lu]Lu-DTPA.

### In vitro autoradiography of [^177^Lu]Lu-DOTA-POL3026

**Fig. 8:**
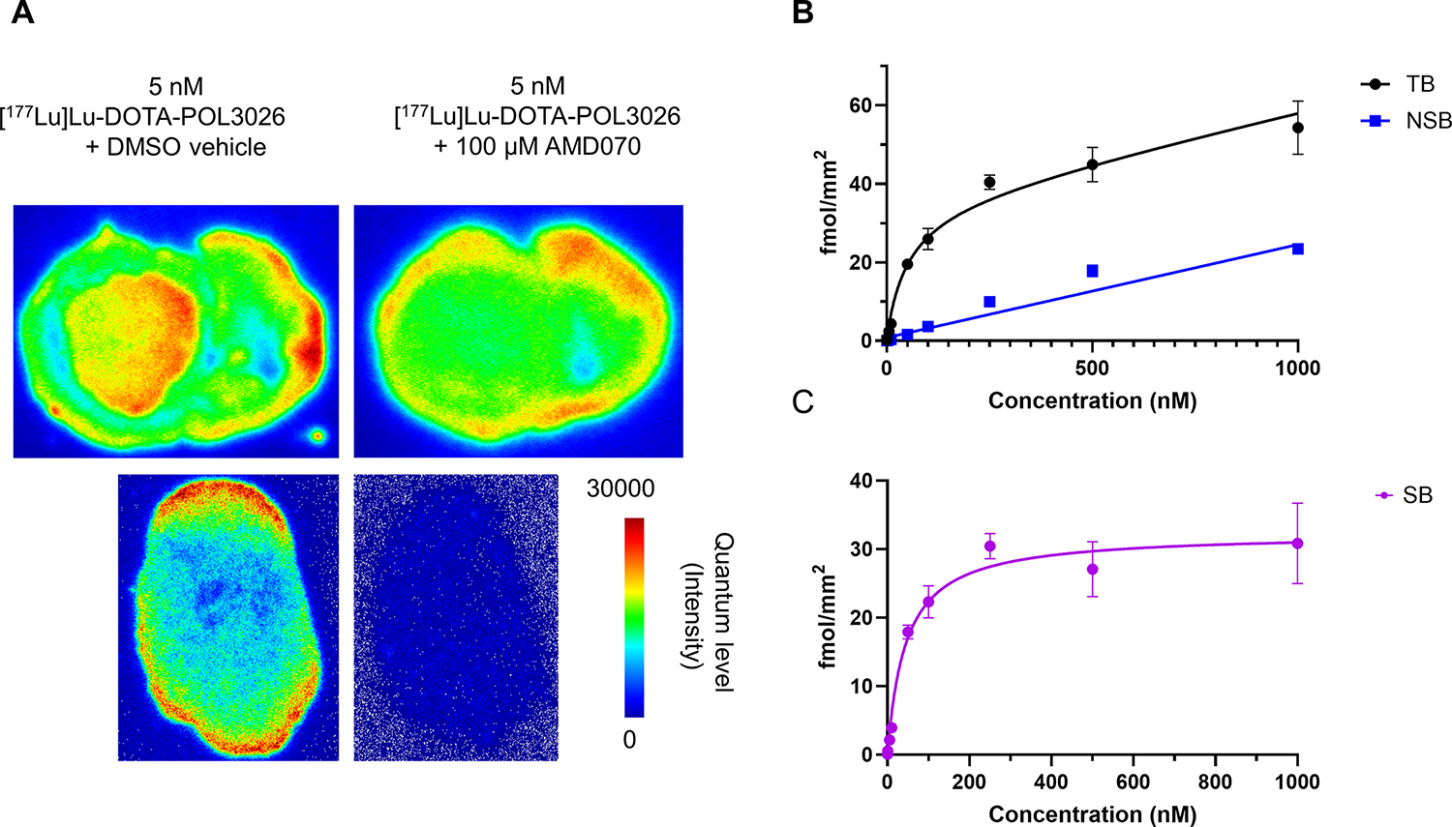
In vitro autoradiography of [^177^Lu]Lu-DOTA-POL3026. The radiolabelled peptide was incubated, in the absence and presence of AMD11070, with coronal sections of a mouse brain with U87-CXCR4 + tumour in the left hemisphere (A, upper panels), and with sections of subcutaneously grown U87-CXCR4 + tumours (A, lower panels). Total binding, non-specific binding (B), and specific binding curve (C) was plotted based on saturation binding assays performed on the U87-CXCR4 + tumour slices. Data expressed as mean ± SD (*N* = 3)

In vitro autoradiography studies were conducted with [^177^Lu]Lu-DOTA-POL3026 on different mouse tissues. To determine binding specificity, sections of subcutaneously grown U87-CXCR4 + tumours and sections of mouse brain with an inoculated U87-CXCR4 + tumour, were incubated with 5 nM [^177^Lu]Lu-POL3026 and treated with vehicle or 100 µM AMD11070 (Fig. 8). High specific binding was observed in the U87-CXCR4 + tumour tissue where up to 90% blocking of [^177^Lu]Lu-DOTA-POL3026 binding was observed. [^177^Lu]Lu-DOTA-POL3026 binding to the U87-CXCR4 + tumour in the mouse brain could be almost completely blocked with an excess of AMD11070. However, non-specific binding was also observed in other parts of the brain.

Binding specificity was similarly evaluated in mouse spleen, with excess AMD11070 resulting in a limited blocking of 11%, indicating predominantly non-specific binding (Supplementary Fig. 2).

Additionally, a saturation binding experiment was conducted on sections of subcutaneous U87-CXCR4 + tumours (Fig. 8). Slices were incubated with [^177^Lu]Lu-DOTA-POL3026 (0.1–1000 nM) in the presence of the vehicle or 100 µM AMD11070 to determine total and non-specific binding respectively. The affinity (K_D_) of [^177^Lu]Lu-DOTA-POL3026 for the U87-CXCR4 + tumour was found to be 52 nM, with a B_max_ of 35 fmol/mm².

### Ex vivo biodistribution of [^177^Lu]Lu-DOTA-POL3026 in tumour-bearing mice and dosimetry estimation

**Fig. 9:**
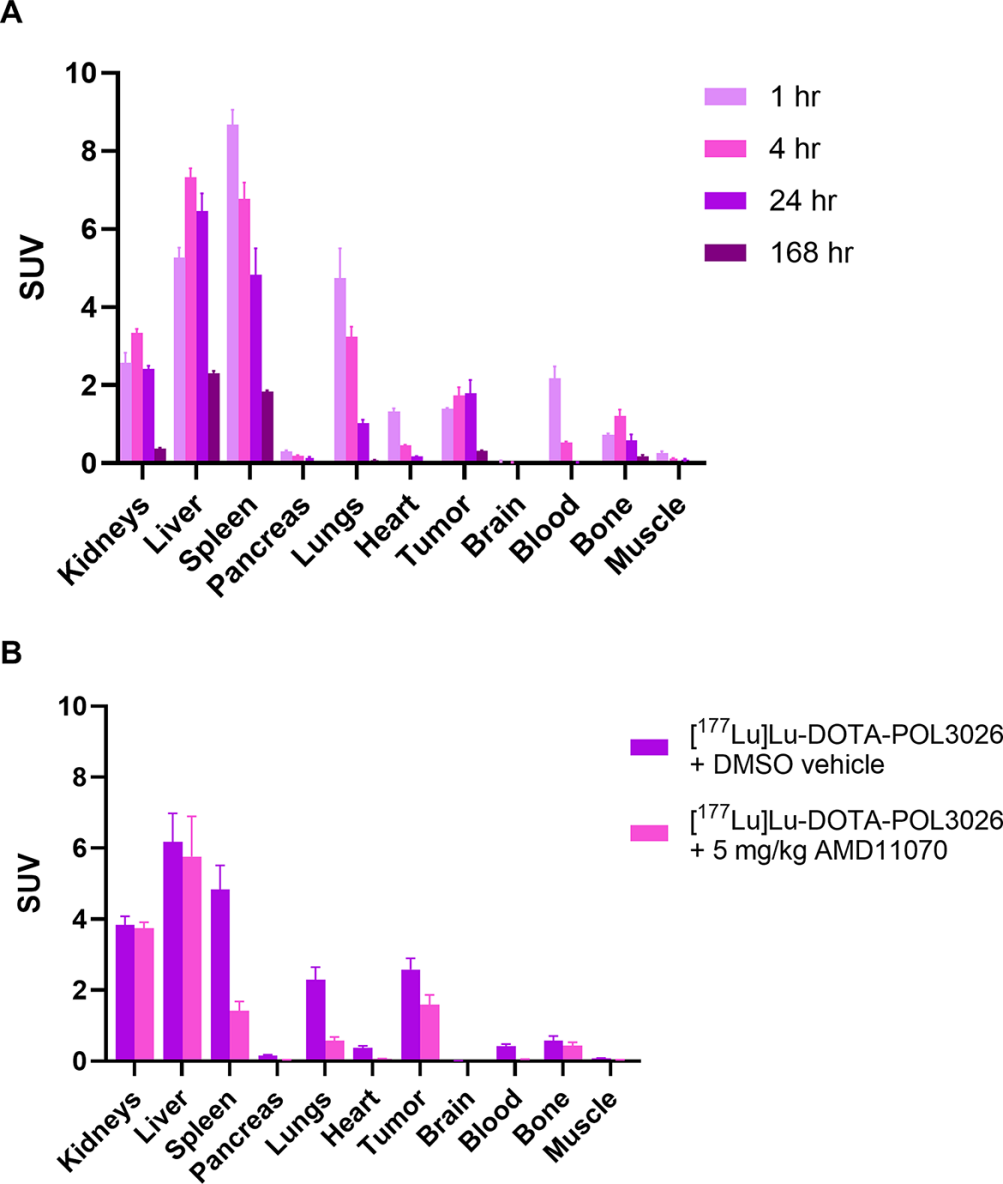
Ex-vivo biodistribution (**A**) and blocking study (**B**) of [^177^Lu]Lu-DOTA-POL3026 in BALB/c nude female mice bearing a subcutaneous U87-CXCR4 + tumour. Activity distributions in organs are expressed as standard uptake value (SUV) depicted as mean + SD (*N* = 3–4)

Biodistribution studies of [^177^Lu]Lu-DOTA-POL3026 were performed in U87-CXCR4 + bearing BALB/c nude mice at 4 time points **(**Fig. 9A**)**. The data is presented as SUV. The clearance of [^177^Lu]Lu-DOTA-POL3026 occurs predominantly via the hepatobiliary pathway. Tumour uptake increased from 1.39 ± 0.04 at 1 h p.i. to 1.79 ± 0.67 at 24 h p.i. The highest uptake was observed in the spleen 1 h p.i. (8.67 ± 0.66), gradually decreasing with time, reaching 1.84 ± 0.04 at 168 h p.i. In the kidneys, the highest uptake was observed at 4 h (3.34 ± 0.19), decreasing to 0.38 ± 0.03 at 168 h p.i. Similarly, the highest uptake in the liver was also observed at 4 h (7.32 ± 0.46), decreasing to 2.3 ± 0.13 at 168 h p.i.

Barely any brain uptake was observed. At 24 h, tumour-to-blood ratio was 89, tumour-to-muscle ratio 19, tumour-to-kidney ratio 0.7, and tumour-to-liver ratio 0.3. Supplementary Tables 1 & 2 show the percentage of injected activity (%IA) values and the SUV values from this experiment.

**Fig. 10:**
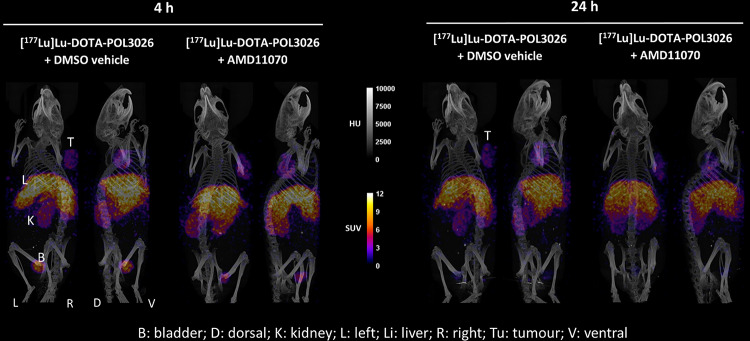
SPECT- CT images shown as maximum intensity projections (MIP) of mice injected with [^177^Lu]Lu-DOTA-POL3026 either with the pre-injection of a DMSO vehicle or AMD11070. Scans were taken 4 h and 24 h post-treatment. The tumour and different organs are indicated with the abbreviated letters clarified at the bottom of the figure

Figure 9B assesses the specificity of [^177^Lu]Lu-DOTA-POL3026 uptake by comparing its uptake at 4 h p.i. in mice that were pre-injected with 5 mg/kg of AMD11070. Results show a 38% reduction in tumour uptake in mice administered with AMD11070 compared to the vehicle group, 71% reduction in spleen, 75% reduction in the lungs, and 7% reduction in the liver. The %IA and SUV values are reported in Supplementary Tables 3 & 4. To confirm the biodistribution trend, µSPECT-CT scans were also performed in the presence or absence of AMD11070 at 4 and 24 h p.i. (Fig. 10). No blocking in the tumour was observed for this study.

The absorbed dose estimated for a spherical tumor (m = 0.16 g) was 0.93 Gy/MBq. For the kidneys, liver and spleen of a mouse model of 25 g the organ absorbed dose was 1.03, 4.70 and 2.2 Gy/MBq, respectively. The organ absorbed dose is predominantly determined by the self-dose component, the cross-dose contribution was 1.2% for the kidneys, 0.02% for the liver and 3.3% for the spleen.

## Discussion

The clinical potential of ^177^Lu-based TRT has been recently highlighted with the FDA and EMA approval of Pluvicto^®^ and Lutathera^®^ for the treatment of mCRPC and GEP-NETs, respectively. This, in turn, has generated considerable interest in the application of ^177^Lu-based TRT for other persistent cancers, including GBM. However, despite the recognised importance of CXCR4 as a therapeutic target in primary and particularly recurrent GBM, research and development of CXCR4-TRT has been sporadic and limited. This is certainly the case for preclinical research that aims to elucidate the underlying in vitro radiobiology of such treatments. Our study aimed to address this gap by preclinically assessing previously reported CXCR4-peptides as potential CXCR4-TRT, focusing on [^177^Lu]Lu-DOTA-POL3026 for more extensive in vitro therapeutic evaluations and in vivo biodistribution studies. We also elucidate the requirements and limitations of such treatments in preclinical models of GBM.

Despite the interest in using CTCE-9908 as a CXCR4 inhibitor for its anti-tumoural, anti-angiogenic, and anti-metastatic effects in different cancer preclinical models, the peptide has never been radiolabelled before (Gravina et al. 2015; Huang et al. 2009; Drenckhan et al. 2013). DOTA was conjugated to the ε-amino moiety of one of the two N-terminal lysines of the dimeric peptide CTCE-9908 (Fig. 1), which may have interfered with its binding to CXCR4, and reduced its affinity. The unconjugated peptide itself was reported to compete with CXCL12 binding, with a half-maximal inhibitory concentration (IC_50_) of around 19.1 ± 2.3 µM in Jurkat cells. DV1-K-DV3 has been investigated as an imaging agent ([^18^F]AlF-NOTA-DV1-K-DV3 and [^68^Ga]Ga-DOTA-DV1-K-DV3) and as a therapeutic agent ([^177^Lu]Lu-DOTA-DV1-K-DV3) (Luyten et al. 2021). In this same study, DOTA-DV1-K-DV3 was found to have an IC_50_ of 27.3 ± 2.8 nM and [^nat^Lu]Lu-DOTA-DV1-K-DV3 an IC_50_ of 2.9 ± 0.6 nM in a competitive binding assay with CXCL12^AF647^ in Jurkat cells. In contrast, results from our saturation binding study in U87-CXCR4 + cells show DV1-K-DV3 to have a low affinity with a K_D_ value in the low micromolar range (± 1 µM), resulting in lower binding at the incubated concentrations. [^177^Lu]Lu-DOTA-POL3026 showed a K_D_ value of 21.8 nM in our saturation binding assay on U87-CXCR4 + cells, and a K_D_ of 52 nM on U87-CXCR4 + tumour slices, determined by in vitro autoradiography. Meanwhile, the IC_50_ of DOTA-POL3026 in a competitive binding assay with CXCL12-Red in Jurkat cells was reported to be 0.47 nM (Lesniak et al. 2015a, b).

The cell membrane-binding and internalisation study confirms the results obtained in the saturation cell binding study, reflecting the low binding of [^177^Lu]Lu-DOTA-CTCE-9908 and [^177^Lu]Lu-DOTA-DV1-K-DV3 to U87-CXCR4 + cells, compared to [^177^Lu]Lu-DOTA-POL3026. A small portion of [^177^Lu]Lu-DOTA-POL3026 was internalised, as expected of peptide receptor antagonists. Indeed, this has been the case for other radiolabelled antagonists when compared to their agonist analogues (Dalm et al. 2016). Internalisation is not a prerequisite for inducing targeted cellular damage via^177^Lu-labeled carriers (or β- emitters in general), as is evident by the observed effects on cell viability and DNA damage. [^177^Lu]Lu-DOTA-POL3026 was chosen for further in vitro and in vivo evaluations.

The capacity of beta particles to traverse several cellular lengths presents a constraint in conducting cell viability assays using beta-emitters like ^177^Lu, due to the contribution of the unbound fraction to cellular damage. Nevertheless, as demonstrated by our findings, an enhanced binding potential (as is the case for [^177^Lu]Lu-DOTA-POL3026) increases the likelihood of inducing fatal cellular damage and reduced cell viability. This effect can be better observed at lower activity concentrations (1–2 MBq/mL), where the influence of unbound activity is minimal (as observed with [^177^Lu]Lu-DTPA). There was no effect of the unlabelled DOTA-conjugated peptides (“cold” peptides) on cell viability at the equivalent concentrations used for the 20 MBq/mL radiolabelled peptides. Indeed, the low concentrations of carrier molecules required for TRT typically do not induce pharmacological or toxicological effects.

The mechanisms through which TRT causes cell death and tumour control are topics of increasing interest within the radiobiology community, as it is important to recognise that the radiation properties of β^-^-emitters such as ^177^Lu are different from those of external-beam radiotherapy in terms of dose-rate and absorbed dose distribution, and the length of time to which cells are irradiated, with consequently different biological effects and cellular mechanisms (Morris et al. 2021; Khazaei Monfared et al. 2023). Compared to vehicle-treated cells, [^177^Lu]Lu-DOTA-POL3026 resulted in a marked increase in both signals, at levels higher than those merely caused by unbound radioactivity in the medium ([^177^Lu]Lu-DTPA). However, considerable variability in the absolute values of calculated parameters between the experiments may have resulted in the lack of statistical significance observed.

DNA damage is considered the main mechanism through which radiation induces cell damage, although there is increasing awareness of the potential role of other cellular targets as mediators of cancer cell damage in TRT (Aerts et al. 2021). Despite GBM cells being incubated for only 4 h with [^177^Lu]Lu-DOTA-POL3026, an activity-dependent increase in γH2AX foci up to 5 days after treatment was observed. This reflects an accumulation of unrepaired DNA double strand breaks, that is likely caused by continuous cell irradiation by the bound radiolabelled peptides. Interestingly, there were fewer 53BP1 foci formed in response to treatment, with no statistically-significant difference compared to the vehicle condition. This may reflect a difference in the kinetics of both proteins, though not much has been elucidated in the kinetics of the DNA damage response to TRT. Additionally, γH2AX and 53BP1 foci did not always overlap, an observation reported by other researchers evaluating ^177^Lu-based radiopharmaceuticals (Ruigrok et al. 2021). Nonetheless, unrepaired double strand breaks can lead to a series of cellular events triggering cell cycle arrest, cell death and/or genomic instability. It is worth noting that U87 cells are considered to be relatively more radioresistant compared to other GBM cell lines or patient-derived GBM stem-like cell cultures (Taghian et al. 1992).

Upon observing microscopic images of the remaining cells five days post-treatment with [^177^Lu]Lu-DOTA-POL3026, a considerable difference in their size and morphology was noticed, compared to those treated with [^177^Lu]Lu-DTPA *[Supplementary Fig. 1]*. The U87-CXCR4 + cells increase in size and adopt a more heterogenous morphology in the remaining cell population 5–7 days post-treatment, ranging from more amoeba-like morphology to increased fibrous projections. It would be interesting to investigate whether this observation indicates a “radiation-tolerant persister” state as reported by Zhao et al. (Zhao et al. 2023) or polyploid giant cancer cells enriched in response to radiation as reported in some GBM cancer cell lines (Alhaddad et al. 2023; Zhang et al. 2021).

Essential to the success of any TRT is the ability of the radiopharmaceutical to specifically target the cancer with minimal uptake in healthy tissues. The biodistribution of [^177^Lu]Lu-DOTA-POL3026 was studied in U87-CXCR4 + tumour-bearing mice, demonstrating uptake in the tumour with high tumour-to-blood and tumour-to-muscle ratios at 24 h p.i. However, uptakes in the kidneys, liver, lungs, and spleen were even higher. This correlates with the results reported by Lesniak et al. using [^111^In]In-DOTA-POL3026 (Lesniak et al. 2015a, b).

Previous studies have reported high endogenous expression of CXCR4 in the murine liver, leading to the expectation that liver uptake would be target-specific (Burke et al. 2020). However, in our study we observed a low liver blocking effect on mice administered with AMD11070. This may be because the AMD11070 dose used was insufficient to cause a reduction in the specific uptake of [^177^Lu]Lu-DOTA-POL3026. Blocking in peripheral tissues, including muscle and blood, increases the free fraction of [^177^Lu]Lu-DOTA-POL3026 that can be redistributed, of which a significant fraction is taken up and excreted by the excretory organs. This becomes apparent in the blocking study where the excreted fraction increased almost 1.5 times (or 46% increase) compared to mice in the vehicle group (Supplementary Table 4). Indeed, the blocking study by Lesniak et al. used three increasing doses of unlabelled POL3026 as a competitive blocker of [^111^In]In-Lu-DOTA-POL3026, and liver uptake increased for the two lower doses and only decreased at the highest dose (Lesniak et al. 2015a, b).

The high specific uptake observed in the spleen can be explained by the high levels of CXCR4 + expression in murine spleen (Nie et al. 2004). Interestingly, in vitro autoradiography on mouse spleen sections (Supplementary Fig. 2) showed a more limited blocking of 11% in sections incubated with AMD11070 which is yet to be explained. Nonetheless, CXCR4 expression in the spleens of patients diagnosed with different solid cancers varied considerably when measured via [^68^Ga]PentixaFor-PET, making it difficult to translate the significance of high spleen uptake in mouse models. Notably, high [^177^Lu]PentixaTher uptake in the spleen of patients with hematologic disease with malignant infiltrations in the spleen is often therapeutically desirable, but this is not the case for patients with solid tumours like GBM (Hänscheid et al. 2022). There is evidence indicating CXCR4 expression in murine and human lung fibroblasts, explaining the specific uptake seen in the lungs in our study (Li et al. 2020; Dupin et al. 2023).

Additionally, it is interesting to note that 4-^18^ F-T140, another CXCR4-targeting, polyphemusin-II-derived peptide, exhibited off-target binding to red blood cells, which was reduced with the addition of low doses of unlabeled peptides (Jacobson et al. 2010).

Indeed, administering a low dose of the unlabeled POL3026 peptide was shown to reduce uptake in blood, lungs, spleen and other peripheral tissues, without affecting uptake in tumour and kidneys, but resulted in higher liver uptake, which suggests that the high uptake observed in several tissues is partly specific (Lesniak et al. 2015a, b).

In vitro autoradiography showed a total blocking of [^177^Lu]Lu-DOTA-POL3026 uptake in tumour sections incubated with an excess of AMD11070, and ex vivo biodistribution results showed a 38% reduction in tumour uptake for mice administered with 5 mg/kg AMD11070. On the other hand, the µSPECT scans did not show a significant reduction in tumour uptake. Ex vivo biodistribution is considered more sensitive for accurate quantification of organ uptakes. The discrepancy with µSPECT may be explained by lower sensitivity and image resolutions, in addition to uncertainty related to the low number of animals scanned for the latter study.

The dose estimations corroborate the trends seen in the biodistribution study, showing that the liver receives an absorbed dose five times higher than the tumour, the spleen receives a dose 2.3 times higher than the tumour, and the kidney receives a dose similar to that of the tumour (1.1 times higher).

Dose calculations were performed assuming a uniform distribution of the activity within the source tissues. In future studies the microscopic distribution of the radiopharmaceutical within tissues and its effect on the dose calculations could be investigated. The OLINDA software was used for calculating S values for dosimetry estimations, however, it does not account for tumour-to-organ and organ-to-tumor cross doses. Moreover, the calculations were based on the 25 g mouse model of OLINDA, whereas the average weight of the mice used in our studies was 19.5 g, potentially introducing some inaccuracies in the dose calculations.

Importantly, results from our biodistribution study show no uptake of [^177^Lu]Lu-DOTA-POL3026 in the brain reflecting the inability of the radiolabelled peptide to cross the blood-brain barrier due to its physicochemical properties. However, in vitro autoradiography showed non-specific binding to brain tissue, casting doubt on the suitability of DOTA-POL3026 for cerebral applications. This needs to be further verified in vivo in orthotopic models with locoregional administration of the radiolabelled peptide.

Most clinical trials investigating TRT for brain tumours use non-systemic administration to bypass the blood-brain barrier (BBB). Indeed, even though the integrity of the blood-brain barrier (BBB) and blood-tumour barrier (BTB) is often compromised in GBM patients, the extent to which those are altered varies, with clinical evidence suggesting that all GBM patients have tumour regions with intact BBB (Sarkaria et al. 2018).

Strategies to circumvent the BBB and BTB for radiopharmaceuticals that do not cross them include: (1) direct administration into the resection cavity; (2) convection-enhanced delivery, which involves the implantation of a microcatheter into the tumour and applying hydraulic pressure to distribute the targeted radiopharmaceutical into the surrounding infiltrated parenchyma; (3) the use of focused ultrasound (FUS) and circulating microbubbles to increase the permeability of BBB/BTB vessels via targeted ultrasonic waves. Integrating these delivery techniques with preliminary molecular imaging, using the diagnostic analogue of radiopharmaceuticals, could facilitate proper patient selection and tailor treatment planning, potentially mitigating the systemic toxicities expected in CXCR4 TRT. Nonetheless, the implementation of such methods requires specialised expertise only available in a limited number of facilities (Bolcaen et al. 2021; Tolboom et al. 2023).

Limitations of our study include the use of a single GBM cell line, U87-CXCR4+. Although U87-MG cells are among the most used for GBM preclinical studies, they do not represent the complexity and heterogeneity of GBM tumours as patient-derived cells would (Xie et al. 2015). Similarly, an orthotopic tumour mouse model, with locoregional delivery of the TRT would perhaps more accurately recapitulate the clinical scenario of future CXCR4 theranostics used in GBM treatments.

In conclusion, our study aimed to preliminarily evaluate the binding characteristics and effect on cell viability of three different ^177^Lu-radiolabelled peptides, [^177^Lu]Lu-DOTA-CTCE-9908, [^177^Lu]Lu-DOTA-DV1-K-DV3, and [^177^Lu]Lu-POL3026 to be used as potential TRT in a preclinical model of GBM. [^177^Lu]Lu-POL3026 was found to have the most favourable characteristics among the three, and was chosen to further investigate its effect on apoptosis and DNA damage in vitro. We found an increase in Annexin V apoptotic signal that is not statistically-significant, and a statistically-significant activity- and time-dependent increase in yH2AX DNA double-strand break marker in response to [^177^Lu]Lu-POL3026 treatment in U87-CXCR4 + cells. Our biodistribution study was the first to evaluate DOTA-POL3026 radiolabelled with ^177^Lu and found results similar to those previously reported with [^111^In]I-DOTA-POL3026, and other polyphemusin-derived CXCR4-targeted peptides. Given the importance of CXCR4 in GBM recurrent tumours and GSCs, and the clinical benefits seen with ^177^Lu-based TRT in other solid cancers, our study highlights the need to develop effective CXCR4-TRT that can eliminate CXCR4 + GBM tumour cells with minimal damage to non-target tissue.

### Electronic supplementary material

Below is the link to the electronic supplementary material.

Supplementary Material 1

## Data Availability

All data generated or analysed during this study are included in this published article and its supplementary information files.

## Abbreviations

%AA: Percentage of Added Activity
%IA: Percentage of Injected Activity
µSPECT: Micro Single Photon Emission Computed Tomography
53BP1: p53–binding protein 1
CXCR4: CXC Chemokine Receptor 4
DMEM: Dulbecco’s Modified Eagle Medium
DMSO: Dimethyl Sulfoxide
DOTA: Dodecane Tetraacetic Acid
FBS: Fetal Bovine Serum
GBM: Glioblastoma
GSC: Glioblastoma stem–like cells
IP: Intraperitoneal
iTLC: Instant Thin Layer Chromatography
IV: Intravenous
MIRD: Medical Internal Radiation Dose
p.i.: Post–Injection
PBS: Phosphate Buffered Saline
SROSEM: Similarity–Regulated Ordered Subsets Expectation Maximization
SUV: Standardised Uptake Value
TAC: Time–Activity Curve
TRT: Targeted radionuclide therapy
γH2AX: Phospho–H2A histone family member X
