# Supplementary material for: Preclinical evaluation of CXCR4 peptides for targeted radionuclide therapy in glioblastoma
Source: EJNMMI Radiopharm Chem. 2024 Jul 15;9:52. doi: 10.1186/s41181-024-00282-y (PMC11250742; doi:10.1186/s41181-024-00282-y)
Supplement: Supplementary file 1 — Supplementary Material 1 [file 41181_2024_282_MOESM1_ESM.docx]

# Supplementary Material:


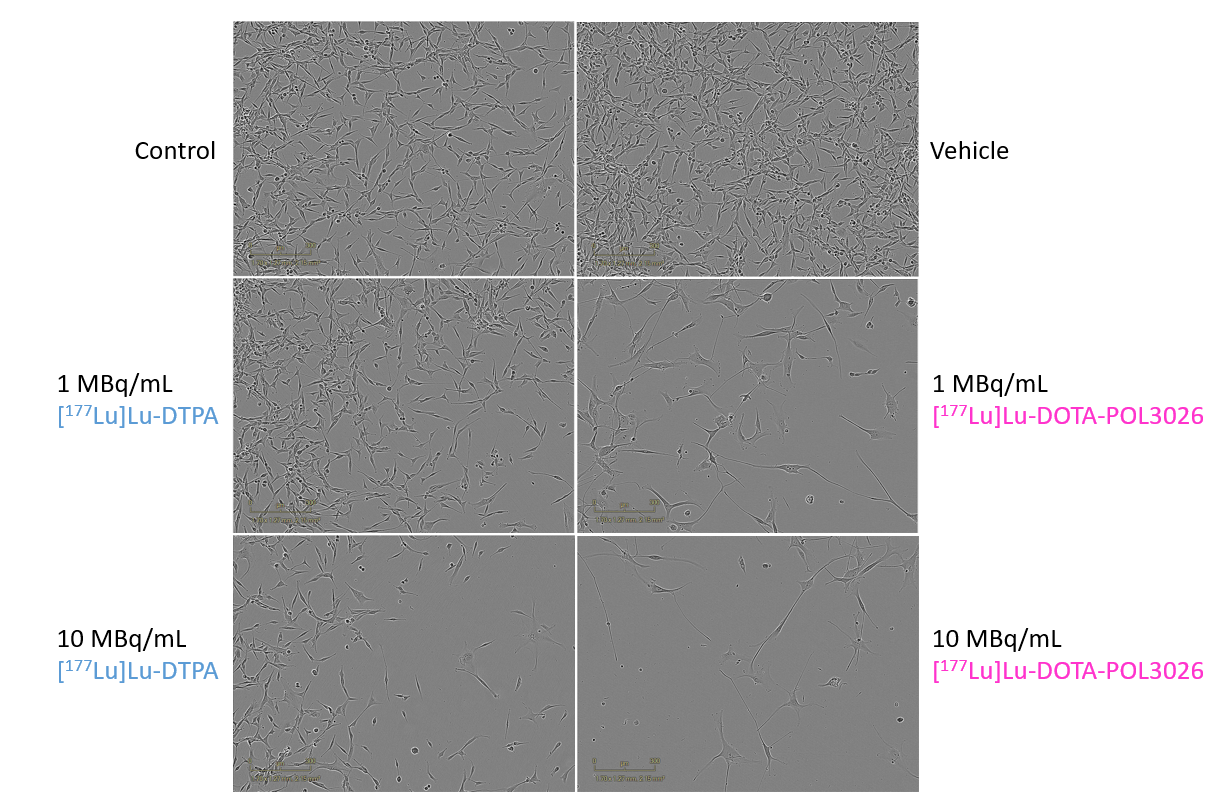


Supplementary Figure 1 – Microscopic images from the IncuCyte live cell imaging system, showing U87-CXCR4+ cells treated with 1 or 10 MBq/mL of [^177^Lu]Lu-DTPA or [^177^Lu]Lu-DOTA-POL3026. Scale bar = 300 µm.


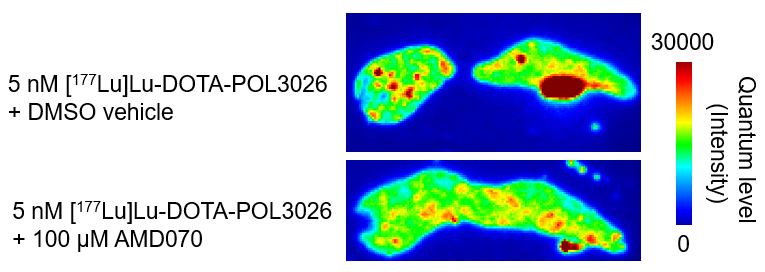


Supplementary Figure 2 – Autoradiography images of mouse spleen sections incubated with [^177^Lu]Lu-DOTA-POL3026 in the absence or presence of AMD11070.

Supplementary Table 1 – Percentage of injected activity (%IA) values of dissected organs from the ex vivo biodistribution study at 1,4, 24, and 168 h p.i. with [^177^Lu]Lu-DOTA-POL3026.

| **%IA** | **1 h** | | **4 h** | | **24 h** | | **168 h** | |
| --- | --- | --- | --- | --- | --- | --- | --- | --- |
| **Organs** | **Mean** | **SD** | **Mean** | **SD** | **Mean** | **SD** | **Mean** | **SD** |
| Kidneys | 4.03 | 0.66 | 4.99 | 0.19 | 3.65 | 0.38 | 0.61 | 0.05 |
| Liver | 31.63 | 1.22 | 39.81 | 1.26 | 39.20 | 1.59 | 14.25 | 0.98 |
| Spleen | 4.01 | 0.88 | 2.02 | 0.54 | 1.61 | 0.25 | 0.72 | 0.05 |
| Pancreas | 0.30 | 0.09 | 0.16 | 0.04 | 0.10 | 0.04 | 0.02 | 0.01 |
| Lungs | 2.72 | 0.64 | 1.87 | 0.12 | 0.59 | 0.01 | 0.06 | 0.01 |
| Heart | 0.71 | 0.15 | 0.22 | 0.01 | 0.09 | 0.01 | 0.01 | 0.00 |
| Intestines | 5.63 | 0.46 | 3.76 | 0.56 | 2.14 | 0.09 | 0.59 | 0.14 |
| Stomach | 0.35 | 0.06 | 0.31 | 0.02 | 0.17 | 0.12 | 0.03 | 0.00 |
| Tumour | 1.54 | 0.05 | 1.01 | 0.49 | 1.46 | 0.53 | 0.29 | 0.12 |
| Brain | 0.09 | 0.05 | 0.03 | 0.01 | 0.01 | 0.00 | 0.00 | 0.00 |
| Carcass | 28.48 | 2.20 | 18.33 | 3.40 | 12.82 | 0.36 | 3.02 | 0.06 |
| Blood on tissue paper | 3.79 | 0.36 | 1.56 | 0.56 | 0.51 | 0.15 | 0.20 | 0.13 |
| Blood | 15.18 | 3.76 | 3.73 | 0.32 | 0.14 | 0.06 | 0.04 | 0.00 |
| Tail | 2.05 | 0.47 | 1.75 | 1.16 | 1.70 | 0.75 | 0.53 | 0.23 |
| Bone | 8.81 | 0.72 | 14.38 | 4.06 | 7.05 | 3.56 | 2.14 | 0.82 |
| Muscle | 9.87 | 4.11 | 4.89 | 0.60 | 3.78 | 1.90 | 0.57 | 0.20 |
| Excreted | 10.82 | 5.76 | 24.23 | 3.99 | 37.50 | 2.18 | 80.16 | 0.78 |

Supplementary Table 2 – SUV values of dissected organs from the ex-vivo biodistribution study at 1, 4, 24, and 168 h p.i. with [^177^Lu]Lu-DOTA-POL3026.

| **SUV** | **1 h** | | **4 h** | | **24 h** | | **168 h** | |
| --- | --- | --- | --- | --- | --- | --- | --- | --- |
| **Organs** | **Mean** | **SD** | **Mean** | **SD** | **Mean** | **SD** | **Mean** | **SD** |
| Kidneys | 2.57 | 0.45 | 3.34 | 0.19 | 2.41 | 0.17 | 0.38 | 0.03 |
| Liver | 5.27 | 0.44 | 7.32 | 0.46 | 6.46 | 0.91 | 2.30 | 0.13 |
| Spleen | 8.67 | 0.66 | 6.77 | 0.83 | 4.83 | 1.34 | 1.84 | 0.04 |
| Pancreas | 0.30 | 0.05 | 0.19 | 0.03 | 0.13 | 0.07 | 0.02 | 0.00 |
| Lungs | 4.74 | 1.32 | 3.24 | 0.51 | 1.02 | 0.19 | 0.08 | 0.01 |
| Heart | 1.33 | 0.13 | 0.46 | 0.02 | 0.17 | 0.02 | 0.02 | 0.00 |
| Tumour | 1.39 | 0.04 | 1.74 | 0.41 | 1.79 | 0.67 | 0.32 | 0.02 |
| Brain | 0.05 | 0.03 | 0.02 | 0.01 | 0.01 | 0.00 | 0.00 | 0.00 |
| Blood | 2.17 | 0.54 | 0.53 | 0.05 | 0.02 | 0.01 | 0.00 | 0.00 |
| Bone | 0.73 | 0.06 | 1.20 | 0.34 | 0.59 | 0.30 | 0.18 | 0.07 |
| Muscle | 0.25 | 0.10 | 0.12 | 0.02 | 0.09 | 0.05 | 0.01 | 0.00 |

Supplementary Table 3 – Percentage of injected activity (%IA) values of dissected organs from the blocking experiment of the ex vivo biodistribution study, with one group of mice pre-injected with DMSO vehicle and another group with AMD11070, prior to the injection of [^177^Lu]Lu-DOTA-POL3026.

| **%IA** | **POL + vehicle** | | **POL + AMD** | |
| --- | --- | --- | --- | --- |
| **Organs** | **Mean** | **SD** | **Mean** | **SD** |
| Kidneys | 5.12 | 0.49 | 5.44 | 0.26 |
| Liver | 33.52 | 7.26 | 33.79 | 14.03 |
| Spleen | 1.74 | 0.57 | 0.51 | 0.22 |
| Pancreas | 0.12 | 0.06 | 0.02 | 0.01 |
| Lungs | 1.23 | 0.35 | 0.35 | 0.15 |
| Heart | 0.16 | 0.03 | 0.03 | 0.01 |
| Intestines | 2.43 | 0.50 | 1.17 | 0.56 |
| Stomach | 0.18 | 0.03 | 0.06 | 0.03 |
| Tumour | 3.35 | 0.33 | 1.68 | 0.63 |
| Brain | 0.02 | 0.01 | 0.01 | 0.00 |
| Carcass | 20.34 | 0.39 | 9.91 | 2.01 |
| Blood on tissue paper | 1.34 | 0.43 | 0.97 | 0.97 |
| Blood | 2.91 | 0.78 | 0.33 | 0.04 |
| Tail | 2.77 | 2.36 | 3.44 | 2.72 |
| Bone | 6.85 | 3.08 | 5.16 | 2.26 |
| Muscle | 3.35 | 0.86 | 1.02 | 0.80 |
| Excreted | 34.49 | 11.09 | 50.41 | 18.17 |

Supplementary Table 4 – SUV values of dissected organs from the blocking experiment of the ex vivo biodistribution study, with one group of mice pre-injected with DMSO vehicle and another group with AMD11070, prior to the injection of [^177^Lu]Lu-DOTA-POL3026.

| **SUV** | **POL + vehicle** | | **POL + AMD** | |
| --- | --- | --- | --- | --- |
| **Organs** | **Mean** | **SD** | **Mean** | **SD** |
| Kidneys | 3.84 | 0.47 | 3.74 | 0.35 |
| Liver | 6.18 | 1.60 | 5.76 | 2.27 |
| Spleen | 4.83 | 1.37 | 1.41 | 0.54 |
| Pancreas | 0.15 | 0.05 | 0.03 | 0.01 |
| Lungs | 2.29 | 0.71 | 0.57 | 0.22 |
| Heart | 0.37 | 0.11 | 0.06 | 0.02 |
| Tumour | 2.58 | 0.63 | 1.59 | 0.55 |
| Brain | 0.01 | 0.01 | 0.00 | 0.00 |
| Blood | 0.42 | 0.11 | 0.05 | 0.01 |
| Bone | 0.57 | 0.26 | 0.43 | 0.19 |
| Muscle | 0.08 | 0.02 | 0.03 | 0.02 |
